# On the Effects of Ethical Climate(s) on Employees’ Behavior: A Social Identity Approach

**DOI:** 10.3389/fpsyg.2018.00960

**Published:** 2018-06-12

**Authors:** Stefano Pagliaro, Alessandro Lo Presti, Massimiliano Barattucci, Valeria A. Giannella, Manuela Barreto

**Affiliations:** ^1^Department of Neuroscience, Imaging and Clinical Sciences, Università degli Studi “G. d’Annunzio” Chieti-Pescara, Chieti, Italy; ^2^Department of Psychology, Università della Campania “Luigi Vanvitelli”, Caserta, Italy; ^3^Università degli Studi eCampus, Rome, Italy; ^4^Department of Psychology, University of Exeter, Exeter, United Kingdom

**Keywords:** ethical climate, organization identification, moral disengagement, organizational citizenship behaviors, counterproductive work behaviors

## Abstract

The spread and publicity given to questionable practices in the corporate world during the last two decades have fostered an increasing interest about the importance of ethical work for organizations, practitioners, scholars and, last but not least, the wider public. Relying on the Social Identity Approach, we suggest that the effects of different ethical climates on employee behaviors are driven by affective identification with the organization and, in parallel, by cognitive moral (dis)engagement. We compared the effects of two particular ethical climates derived from the literature: An ethical organizational climate of self-interest, and an ethical organizational climate of friendship. Three hundred seventy-six workers completed measures of Ethical Climate, Organizational Identification, Moral Disengagement, Organizational Citizenship Behaviors (OCBs), and Counterproductive Work Behaviors (CWBs). Structural equation modeling confirmed that the two ethical climates considered were independently related to organizational identification and moral disengagement. These, in turn, mediated the effects of ethical climates on OCBs and CWBs. We discuss results in light of the social identity approach, and present some practical implications of our findings.

## Introduction

The spread and publicity given to questionable practices in the corporate world during the last two decades (e.g., scandals, frauds, bankruptcies, conflicts of interest, violation of workers’ rights, etc.) have fostered an increasing awareness of, and interest in, the importance of ethical work climates (Victor and Cullen, 1988) for organizations, practitioners, scholars, and the wider public (Barattucci et al., 2017). The growing interest in the study of *ethical climates* – defined as a set of *shared* perceptions of procedures and policies, both formal and informal, which shape expectations for ethical behavior (Victor and Cullen, 1987) – relies on the observation that they strongly influence individual and organizational outcomes and behaviors (Treviño et al., 1998; Ehrhart and Raver, 2014; Mayer, 2014). Specifically, ethical climates that promote pro-social behavior generally tend to be associated with stronger work performance than ethical climates that promote individualism (e.g., Briggs et al., 2012; Ehrhart and Raver, 2014; Mayer, 2014). Thus, the understanding of positive proximal (e.g., job attitudes) and distal (e.g., lowered performance) organizational and individual consequences that different ethical climates may foster, can help organizations harness their potential.

How does ethical climate – or better, how do different types of ethical climates – impact upon individual’s attitudes and behavior? Relying on the Social Identity Approach (Tajfel and Turner, 1979; Turner et al., 1987), in the present paper we suggest that the effects of different ethical climates on employees’ behavioral tendencies are driven by affective identification with the organization and, in parallel, by cognitive moral (dis)engagement (Bandura, 1990). Knowing whether and how different ethical climates foster organizational identification and moral disengagement could be helpful to tailor organizational interventions targeting specific dimensions of organizational climate, to foster positive organizational identification, or to discourage moral disengagement. In particular, we aim to show that different kinds of ethical climates are related to different levels of organizational affective identification and cognitive moral (dis)engagement toward the organization as a whole, which impact on the employees’ positive (i.e., Organizational Citizenship Behavior, hereafter *OCB*; e.g., Brief and Motowidlo, 1986) and negative (i.e., Counterproductive Work Behaviors, hereafter *CWB*; e.g., Spector and Fox, 2005) behavioral reactions.

### Ethical Climate(S)

An ethical climate can be referred to as “the perception of what constitutes right behavior, and thus becomes a psychological mechanism through which ethical issues are managed” (Martin and Cullen, 2006, p. 177). As such, the construct of ethical climate approaches the notion of moral norms, that is, behavioral guidelines that drive the interpretation of what is right and wrong within groups and organizations (see for instance, Ellemers et al., 2008; Pagliaro et al., 2011). This refers to the fact that different organizations develop different subcultures that govern how individuals relate to each other and regulate each other’s behavior. Starting from the original ethical climate theory (Victor and Cullen, 1987, 1988), a growing body of research has focused on the consequences of ethical climate on employees’ perceptions and behaviors. In this vein, researchers ascertained that ethical climates predict employees’ ethical behaviors (Verbeke et al., 1996; Treviño et al., 1998), job attitudes (e.g., job satisfaction; Deshpande, 1996; Schwepker, 2001; Ambrose et al., 2008), commitment to the organization (e.g., Treviño et al., 1998; Babin et al., 2000), turnover intentions (Schwepker, 2001; Mulki et al., 2006; Ambrose et al., 2008), OCBs (Leung, 2008; Shin, 2012), and a range of CWBs (for a review, see Ehrhart and Raver, 2014; Mayer, 2014).

A second important area of investigation around ethical climate has focused on the understanding of the different facets of the concept itself. While some have regarded it as a single construct (see Schwepker, 2001; Arnaud and Schminke, 2007; Mayer, 2014), the concept of ethical climate is more commonly regarded as multi-dimensional (Victor and Cullen, 1988; Babin et al., 2000; DeConinck, 2011). For example, in their seminal work Victor and Cullen (1987, 1988) proposed a theoretical typology of ethical climates comprising nine ethical climate types based on two dimensions: (a) the criteria used for making ethical decisions (i.e., egoism, benevolence, and principles) and (b) the locus-of-analysis (i.e., individual, local, and cosmopolitan). The following from this proposal, several types of ethical climates have been theorized, as well as different ways of differentiating between them. An important distinction for the present purpose can be made between an ethical organizational climate of *self-interest*, which underlines a more individualistic and independent way of dealing with ethical issues within the organization; and an ethical organizational climate of *friendship*, which, on the contrary, subsumes a collective and interdependent way to deal with the same ethical issues (Cullen et al., 1993). In the present paper, we deal in particular with the relationship between these two ethical climates and employees’ positive and negative behaviors, in terms of OCBs – that is, discretionary behaviors that are favorable to the organization (Brief and Motowidlo, 1986) – and their extreme opposite, CWBs – that is, “volitional acts that harm or intend to harm organizations and their stakeholders” (Spector and Fox, 2005, pp. 151–152).

Although both climates of friendship and self-interest may have positive effects, when it comes to promoting pro-organizational behavior like OCB (and/or to discourage negative tendencies such as CWBs), we propose that an ethical climate of friendship (vs. self-interest) will have the upper hand. This is because such an interdependent and collective way of dealing with ethical issues within organizations is more likely to promote organizational affective identification, and discourage cognitive moral disengagement. The relation between ethical climate, on the one hand, and OCB (e.g., Leung, 2008; Shin, 2012) and a plethora of CWBs (for a review, Ehrhart and Raver, 2014) on the other, has been demonstrated in the literature: however, far less attention has been devoted to the investigation of the affective and cognitive mechanisms that underlie such relation. Clarifying these mechanisms is the main goal of the present paper.

### Organizational Identification and Its Relationship With Ethical Climate

The seminal work on organizational identification by Ashforth and Mael (1989) highlighted the fact that applying the social identity approach (Tajfel and Turner, 1979; Turner et al., 1987) to organizations allowed a new understanding of many organizational dynamics (e.g., leadership; conflict and conflict resolution; job strain amongst others). The central statement of the social identity approach (Tajfel, 1978) is that, whereas in many situations people think about themselves as unique and independent individuals, who behave on the basis of their own idiosyncratic characteristics, in many other contexts they are inclined to think of themselves (and others, in turn) in terms of group membership (e.g., in terms of their belonging to an organization). Most importantly, in the cognitive elaboration of the approach – that is, self-categorization theory – Turner et al. (1987) elaborated on the conditions under which different levels of self-definitions (personal vs. social) are likely to become salient, as well as on the perceptive and behavioral consequences of those different self-definitions (Oakes et al., 1994; see also, Ellemers et al., 2004). Thus, the social identity approach specifies how group membership – and the affective connection with one’s group (i.e., social identification) – provides individuals with normative guidelines that help them define who they are, how to behave, and which aspects of their group belongingness are particularly important (Ellemers et al., 2013). As a result, their group belongingness becomes part of their collective self-concept.

Building on Ashforth and Mael (1989, 1996), researchers highlighted the link between organizational identification and several aspects of organizational life (see Riketta, 2005, for a review), including, for instance, work motivation (Haslam et al., 2000), turnover intentions (Ellemers et al., 2011; Smith et al., 2012; Ngo et al., 2013), organizational trust (Roeck and Delobbe, 2012), procedural justice (Epitropaki, 2013), perceived organizational support (Cheung and Law, 2008), and job performance (Riketta, 2005). Nevertheless, only few studies have tried to connect organizational identification and ethical climate (Umphress et al., 2010; DeConinck, 2011; Walumbwa et al., 2011; Briggs et al., 2012; DeConinck et al., 2013). Among these, the most relevant for the present purpose is the work by DeConinck (2011), who found that facets of ethical work climates (i.e., responsibility/trust) were directly related to organizational identification in a sample of salespeople. Nevertheless, this previous research did not investigate whether or not different ethical climates relate to organizational identification, nor has it ascertained how this further impacts upon employees’ positive and negative organizational behaviors, as we tried to do in the present research. The lack of empirical studies on the relation between ethical climate and organizational identification is at odds with recent evidence of how central moral/ethical issues are for group belongingness and identification (DeConinck, 2011; Pagliaro et al., 2011; Ellemers et al., 2013; van Prooijen and Ellemers, 2015). In fact, individuals prefer to identify with, and are particularly proud of, groups and organizations that are considered as moral and honest (for a review, Ellemers et al., 2013), and this centrality of organizational morality leads them to commit themselves to the organization (Ellemers et al., 2011). Following this rationale, it should be predicted that strong ethical climates facilitate employee organizational identification (Allen and Meyer, 1990; Becker et al., 1996), in particular when this subsumes a collective and interdependent (vs. an individual and independent) way to behave within the organization. This is why in the present research we focused on the comparison between an ethical organizational climate of *self-interest* and an ethical organizational climate of *friendship*, which subsume two opposite ways to deal with the same ethical issues (Cullen et al., 1993).

### Moral Disengagement in Organizational Contexts

Bandura (1990, 1991) introduced the concept of moral disengagement to identify several social-cognitive mechanisms that people use to justify or rationalize their wrongful and deviant antisocial behaviors. In particular, it is posited that individuals generally behave in ways that are consistent with their internal standards of morality because they may experience anticipatory negative emotions (e.g., remorse, guilt, and shame) when they deviate from those internal standards. However, under specific situations individuals might perform behaviors that violate such internal standards, even when these remain unchanged, thus effectively bypassing the negative emotions that would normally emerge from such violation. Moral disengagement is therefore a process that allows individuals to regard their negative behavior, and its consequences, in a socially acceptable way (Gibbs et al., 1995). It can operate as an automatic and anticipatory mechanism preventing individuals from perceiving moral inconsistencies, or as a *post hoc* rationalization (Bandura et al., 1996, 2000; Ashforth and Anand, 2003; Martin et al., 2014; Fida et al., 2015). While some scholars focused on moral disengagement as a disposition (e.g., Moore et al., 2012), others have started to examine how situational or contextual factors may promote moral disengagement and unethical behaviors (Kish-Gephart et al., 2014; Martin et al., 2014).

In organizational contexts, researchers have ascertained the relationship between moral disengagement on the one hand and a number of misconducts on the other hand, including violations of legal and moral rules in the production process (Brief et al., 2001), unethical work behaviors (Moore, 2008; Barsky, 2011), and violation of safety rules (Barbaranelli and Perna, 2004; for a review, see Treviño et al., 2006). Deviance can have profound negative effects on organizational performance and can be very costly (Huang et al., 2017). Ethical climates might influence the self-regulation process that reduces or increases negative behaviors, allowing workers to keep a positive view of themselves even if they undertake deviant actions (Christian and Ellis, 2014; Baron et al., 2015; Greenhalgh et al., 2015).

Although the relationship between moral disengagement and CWBs has been ascertained, far less attention has been devoted to the possible relationship between ethical climate and moral disengagement. Saidon et al. (2012) found that moral disengagement mediated the effect of ethical climate on interpersonal deviance. Nevertheless, as noted above, ethical climate has been acknowledged as a multi-dimensional construct, which underpins different ways of treating moral issues within organizations. Later, Paharia et al. (2013) found that situations promoting self-interest fostered moral disengaged reasoning and behaviors. Thus, we are inclined to believe that the specific (individual and independent vs. collective and interdependent) nature of the ethical climate endorsed within an organization may favor moral disengagement toward that organization and subsequently promote anti-social behaviors. In particular, we suggest that the more the organization endorses a collective and interdependent way to treat moral issues (i.e., a friendship ethical climate), the fewer employees should morally disengage, the more they should display OCBs and the less they should display CWBs.

### The Present Research

In the present research we directly tested how two different kinds of ethical climate relate to employees’ affective identification with organization, cognitive moral disengagement, and (positive and negative) behaviors. In particular, considering that social identity approach stresses the importance of the psychological link with other ingroup members, we considered two particular ethical climates: An ethical organizational climate of self-interest, which underlines an individualistic way to deal with ethical issues within the organization, and an ethical organizational climate of friendship, which on the contrary subsumes a more collective and interdependent way to deal with the same ethical issues (Cullen et al., 1993). Our main proposition is that the more the ethical climate of an organization endorses a collective way to manage the ethical issues, the more employees should be encouraged to strengthen their psychological linkage with their peers and their organization as a whole, and the less they should therefore disengage from their own organization. As a result, they should behave in a way that benefits the organization as a whole rather than themselves as individuals. On the contrary, the more the ethical climate of an organization promotes individuality, the fewer employees should be encouraged to strengthen their psychological linkage with their peers and their organization as a whole, and the more they should consider correct to behave in an individualistic way, even in ways that threaten the organization as a whole. As a result, they should not invest time and energies in behaviors that benefit the organization as a whole.

**FIGURE 1:**
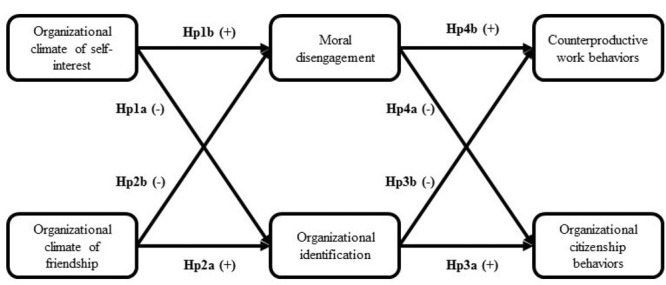
Research model and hypotheses.

In summary, we anticipated that the perception of an ethical organizational climate of self-interest would be negatively related to organizational identification (Hp1a) and positively related to moral disengagement within the organization (Hp1b). On the contrary, we anticipated that the perception of an ethical organizational climate of friendship should be positively related to organizational identification (Hp2a) and negatively related to moral disengagement within the organization (Hp2b). Moreover, we hypothesized that both organizational identification and moral disengagement should be related to employees’ positive and negative behaviors, in terms of OCB and CWB, although in opposite directions. That is, organizational identification should be positively related to OCB (Hp3a) and negatively related to CWB (Hp3b). On the contrary, moral disengagement should be negatively related to OCB (Hp4a) and positively related to CWB (Hp4b). Finally, in order to clarify whether the effects of ethical climate on OCB and CWB are driven by organizational identification and moral disengagement, we tested whether these effects are actually mediated by both organizational identification and moral disengagement (Hp5). We tested these hypotheses in a comprehensive structural equations modeling. **Figure 1** depicts our research model and summarizes the main hypotheses.

## Materials and Methods

### Participants and Procedure

Data were collected among employees at eleven Italian small and medium enterprises (SMEs) in different sectors: insurance, health, chemical, manufacturing, and welfare. We adopted this sampling procedure because SMEs are the typical Italian organization and this could help achieve the highest variability in responses.

The final sample consisted of 376 workers, 215 (57.2%) of whom were women (9 missing cases). Mean age was 40.35 years (SD = 10.95), while average organizational tenure was 10.98 years (SD = 10.69). One hundred twenty-seven respondents (33.8%) were single, 212 (56.4%) were married/cohabitating and 18 (4.8%) were divorced or widowed (19 missing cases). 26 (6.9%) held a junior high school degree or less, 161 (42.8%) a high school degree and 175 (46.5%) a university degree (14 missing cases). As for employment contract, 258 (68.7%) held a permanent contract against 98 (26.1%) with a permanent contract (20 missing cases).

Respondents received a copy of the questionnaire, along with a letter of research presentation and a sealable envelope in order to protect privacy (Italian Law No. 196/2003). Questionnaires were distributed within organizations by trained researchers and participation was voluntary. Researchers contacted the human resource managers of the companies, explained the topic under investigation to them, and these collectively distributed the questionnaires to the employees. Employees were asked to fill in the questionnaires and return them within 2 weeks. Individuals had the opportunity to stop their participation at any time. The study complied with ethical standards and was approved by the Ethical Committee of the ALP institution.

### Measures

*Ethical organizational climate of self-interest* (Cullen et al., 1993; Italian version: Lo Presti et al., 2017) was assessed through four items (e.g., “In this company, people are mostly out for themselves”). Responses were given through a 6-point scale (from 0 = “completely false” to 5 = “completely true”; Cronbach’s alpha = 0.75).

*Ethical organizational climate of friendship* (Cullen et al., 1993; Italian version: Lo Presti et al., 2017) was assessed through six items (e.g., “In this company, people look out for each other’s good”). Responses were given through a 6-point scale (from 0 = “completely false” to 5 = “completely true”; Cronbach’s alpha = 0.90).

*Organizational identification* was assessed through the Italian adaptation by Manuti and Bosco (2012) of the original 6-item scale by Mael and Ashforth (1992), adapted for organizational contexts (e.g., “When someone criticizes my organization, it feels like a personal insult”). Responses were given on a 6-point Likert scale (from 0 = “completely disagree” to 5 = “completely agree”; Cronbach’s alpha = 0.92).

*Moral disengagement* was evaluated through the Work Moral Disengagement Scale (Fida et al., 2015). The scale comprised 24 items (e.g., “Being absent from work frequently is acceptable since many people at work are not productive anyway”) through a 6-point Likert scale (from 0 = “completely disagree” to 5 = “completely agree”; Cronbach’s alpha = 0.93).

*Counterproductive work behaviors* were assessed through the Italian version (Barbaranelli et al., 2013) of the original checklist by Spector et al. (2006). In particular, we assessed CWB toward organizations, that is behaviors targeting the organization as a whole, through 13 items (e.g., “stole something belonging to my employer”). Responses were collected via a 6-point frequency scale (from 0 = “never” to 5 = “always”; Cronbach’s alphas = 0.95).

Finally, to assess *OCB* we administered the Italian version (Argentero et al., 2008) of the original questionnaire by Podsakoff et al. (1990). The scale includes 15 items (e.g., ‘Help others who have heavy workloads’). Participants responded by using a 7-point frequency scale (from 1 = ‘never’, to 7 = ‘always’; Cronbach’s α = 0.97).

### Data Analysis

Structural equation modeling analysis (Lisrel 9.3) using Maximum Likelihood estimation methods (along with the indicators’ covariance matrix) were used to evaluate the measurement and structural models concerning study variables and their associations.

With regard to the measurement model, given the low ratio (5.52) between cases (i.e., 376) and the number of items (i.e., 68) we relied on the item parceling technique (Little et al., 2002), which allows parcel creation on the basis of the calculated mean between different items referring to the same construct. Advantages of this technique include fewer instability risks in the parameters’ estimates, fewer estimation problems deriving from the small number of cases, and fewer risks deriving from items’ non-normality or the excessive number of parameters to be estimated.

Item parceling was used for the following variables: moral disengagement (6 items for 24 items), CWBs (6 parcels for 13 items), OCBs (5 parcels for 15 items), increasing the cases/item ratio to 11.39.

With regard to the models’ goodness-of-fit evaluation, we relied on both absolute and relative goodness-of-fit indices. In addition to referring to the chi-square and degrees of freedom to evaluate possible significant differences between alternative nested models, we reported the following indices: root mean square error of approximation (RMSEA; acceptable values lower than 0.08; Browne et al., 1993), standardized root mean square sesidual (SRMR; acceptable values lower than 0.08; Hu and Bentler, 1999), comparative fit index (CFI) and incremental fit index (IFI), for which scores higher than 0.90 are acceptable (Hoyle, 1995; Marsh et al., 1996).

Given that we measured all study variables through a single questionnaire, we addressed common method variance and response bias according to methods outlined by Podsakoff et al. (2003). Different scale endpoints and formats for the predictor and criterion measures were used in order to reduce method biases caused by commonalities in scale endpoints and anchoring effects. Moreover, we randomly inserted items into the questionnaire and scales were graphically separated from each other. Finally, two different versions of the questionnaire, containing a different scales’ sequence, were used for data collection.

## Results

**Table 1** reports the descriptive statistics and zero-order correlations among the variables of the study.

**Table 1:** Descriptive statistics and zero-order correlations among the variables of the study.

	M (*SD*)	1	2	3	4	5	6
(1) Organizational climate friendship	16.15 (*7.91*)						
(2) Organizational climate self-interest	11.94 (*5.08*)	–0.41***					
(3) Organizational identification	2.88 (*1.38*)	0.34***	–0.29***				
(4) Moral disengagement	0.84 (0*.80*)	–0.15**	0.21***	–0.10*			
(5) Organizational citizenship behaviors	5.65 (0.*98*)	0.30***	–0.20***	0.30***	–0.39***		
(6) Counterproductive work behaviors	0.33 (0.*73*)	0.02	0.10	–0.22***	0.36***	–0.25***	

*^∗∗∗^*p* < 0.001, ^∗∗^*p* < 0.01, ^∗^*p* < 0.05.*

A measurement model was developed in order to examine the construct validity of study measures using confirmatory factor analysis (CFA). A common method is to compare different models (nested models), from a one-factor model to a final one containing as many factors as included measures (in our case six latent variables), passing over intermediate solutions. The different models were compared on the basis of chi square/degrees of freedom scores, and on different goodness of fit indices.

Three different measurement models were developed: A one single factor model, a three factors model (predictors, mediators, outcomes), and finally the model to be confirmed, containing six separate factors (**Table 2**).

**Table 2:** Alternative measurement models on study variables, including goodness of fit indices.

	Chi square	df	RMSEA	CFI	IFI	SRMR
Model 1 – one factor	6952.35	495	0.186	0.338	0.340	0.203
Model 2 – three factors	4389.71	492	0.145	0.600	0.602	0.180
Model 3 – six factors	1199.84	480	0.063	0.926	0.927	0.0507

There was a remarkable improvement from model 1 to model 3, demonstrated by all the included indices. Such results supported a measurement model in which items and parcels referred to their respective latent variables making it possible to estimate the subsequent structural model. We subsequently verified scales’ internal consistencies through Cronbach alphas. Cronbach alpha values were satisfactory for all scales (see section “Measures”).

**FIGURE 2:**
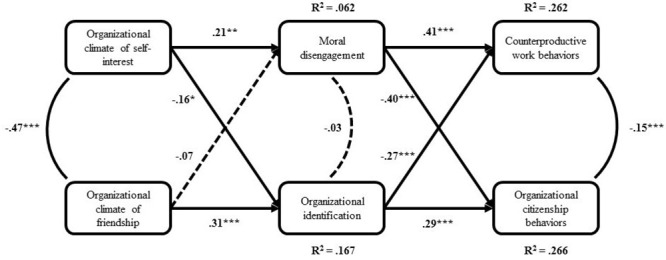
Structural model on study variables.

The subsequent structural model contained all hypothesized relationships (see **Figure 2**): direct links from the two predictors (organizational ethical climates of self-interest and friendship) to organizational identification and moral disengagement as intermediate and mediating variables, while these two latter variables linked to CWBs and OCBs as outcomes.

Such model (χ^2^= 1225.58, *df* = 484) showed acceptable goodness of fit indexes: RMSEA = 0.064, CFI = 0.924, IFI = 0.924, SRMR = 0.0604, and being consistent with our hypotheses and research model, was retained. Organizational ethical climate of self-interest was negatively related to organizational identification (β = -0.16, *p* < 0.05; Hp1a) and positively related to moral disengagement (β = 0.21, *p* < 0.01; Hp1b), while friendship climate was only positively related to organizational identification (β = 0.31, *p* < 0.001; Hp2a). Contrary to Hp2b, friendship climate was not reliably associated with moral disengagement, although the relationship between these variables was in the predicted direction. Thus, our data seems to suggest that friendship climate impacts more on positive (i.e., identification) than negative (i.e., disengagement) reactions. Organizational identification was positively related to OCBs (β = 0.29, *p* < 0.001; Hp3a) and negatively related to CWBs (β = -0.27, *p* < 0.001; Hp3b); a reverse pattern emerged concerning moral disengagement, which was negatively related to OCBs (β = -0.40, *p* < 0.001; Hp4a) and positively related to CWBs (β = 0.41, *p* < 0.001; Hp4b). Organizational ethical climates of self-interest and friendship showed a significant correlation (*r* = -0.47, *p* < 0.001), as well as CWBs and OCBs (*r* = -0.15, *p* < 0.001), while moral disengagement and OCBs did not show any reciprocal correlation. Moreover, with regard to the indirect effects, according to Hp5 our proposed mediators (i.e., organizational identification and moral disengagement) totally mediated the effects of self-interest climate on CWBs (indirect β = 0.13, *p* < 0.01), and OCBs (indirect β = -0.13, *p* < 0.01), as well as the effects of friendship climate on CWBs (indirect β = -0.11, *p* < 0.01) and OCBs (indirect β = 0.11, *p* < 0.01). Finally, the links inserted in the model explained the following variances for dependent variables: Moral disengagement = 6%, organizational identification = 17%, CWBs = 26%, OCBs = 27%.

## Discussion

In the present paper we dealt with the impact of ethical climate on employees’ behavior. Relying on the Social Identity Approach, we proposed that the effects of different ethical climates on employees’ behavioral tendencies may be driven by a process of affective identification with one’s organization and, in parallel, by cognitive moral disengagement toward the organization itself. Our findings showed that an ethical organizational climate of self-interest, which underlines an individualistic way of dealing with ethical issues within the organization, reduces organizational affective identification and enhances a cognitive process of moral disengagement. This, in turn, facilitates CWBs and inhibits OCBs among employees. Conversely, an ethical organizational climate of friendship, which subsumes a collective way of dealing with the same ethical issues, strengthens affective identification with the organization: This produces more tendencies to OCBs and reduces CWBs among employees. Thus, our findings suggest that the effects of ethical climate on individual’s behavioral reactions are driven by the psychological link with the organization (see also DeConinck, 2011), and point to the fact that different ethical climates impact differently on organizational identification and moral disengagement. In this way, the present research contributes to the literature by showing the positive association between organizational identification and employees’ attitudes and behaviors (Ashforth and Mael, 1989, 1996; Haslam et al., 2000; Riketta, 2005; Ellemers et al., 2011; Smith et al., 2012), and provides support to the idea that ethical climates relate to different levels of organizational identification (DeConinck, 2011; Briggs et al., 2012; Walumbwa et al., 2011; DeConinck et al., 2013).

The present research also shows that organizational identification and moral disengagement play their roles in a rather independent way. This result has a strong applied value, since it suggests that organizational identification is not an antecedent of moral disengagement. Moreover, the fact that the ethical climate of friendship was not reliably associated with moral disengagement is theoretically significant too, suggesting that different facets of ethical climate affect employees’ reactions for different reasons (identifying with one’s own organization on the one hand, morally disengaging from it on the other hand).

Particular attention should be devoted to an ethical climate based on self-interest, individualism and competitiveness, because our results suggest that it may negatively influence identification with the organization and lead to morally disengage from the organization: Thus, recognizing individualistic tendencies in ethical climate appears crucial for the organization.

### Practical Implications

Modern working environments can be profitably understood through ethical context and morality terms. Many companies are currently engaged in the contrast of unethical behaviors of their own managers and employers, and they are increasingly stimulated to be guided by ethical principles and environments. Institutions, organizations and magazines started to watch over companies’ ethical behavior or offering ethical code advice and consulting.

Our results suggest some general guidelines for HR staff and managers. First, companies are called to pay more attention to developing adequate ethical infrastructures, in order to reduce their effects on moral disengagement processes and its subsequent behaviors: Organizations need to strengthen those processes that subsume the development of positive ethical cultures and climates. More specifically, in spite of organizational efforts to use and practice ethical codes, a competitive context and culture may be responsible for counterproductive behavior. When formal (e.g., reward systems) and informal (e.g., rumors) signals spread the organization and prompt that individualistic, result-oriented, aggressive practices are recommended, good codes, and regulations may not work properly.

Furthermore, it has become increasingly clear that assessing ethical climate in organizations is critical and that it should be done periodically, in order to share and discuss results and perceptions together. HR managers should be aware that the communication of the results may have a critical impact on employee’s perceptions and thus influence other organizational outcomes. Based on our findings, organizations would also benefit from understanding how identification can be enhanced, as a powerful tool to engage individuals within the organization, suggest how to recognize signals and antecedents of ethical problematical situations, and lead to proper solutions.

Perceptions of ethical climate are a basic requirement for the effective functioning of organizations and may influence several outcomes (e.g., Schwepker, 2001; Cullen et al., 2003; Tanner et al., 2015). Ethical climate becomes a strategic variable for the efficient overall operation of the company, commitment, reward system, corporate identity construction processes and organizational citizenship (Ostroff et al., 2002; Schneider et al., 2011).

### Limitations and Future Directions

Even though our findings support our main hypotheses, the present research presents some limitations that need to be addressed in future studies. First, we compared two specific ethical climates that, in our opinion, could lead the interviewed employees to differentially identify with the organization and, in contrast, to show moral disengagement. Although, based on the social identity approach, we chose to compare an individualistic and independent (i.e., self-interest) with a collectivistic and interdependent (i.e., friendship) ethical climate, a natural extension of the present research would be to check how the other facets of ethical climate impact upon employees’ tendencies to broaden their collective self to incorporate core aspects of the organization they belong to. Moreover, it is worth nothing that we assessed perceptions of ethical climate as an individual-level variable, as abundant literature did, but future research could also consider ethical climate as a contextual variable beyond the perceptions of employees.

A further extension would be to consider other outcomes of the processes we highlighted. Here we focus on behavioral positive and negative tendencies, in the form of OCBs and CWBs, respectively. It would be interesting to consider how the different facets of ethical climate influence employees’ wellbeing – in terms of stress or empowerment perception – through organizational identification. According to the social identity approach, this could be particularly important when employees compare their own organization (that is, their ingroup) with what happens in a competitor context (that is, their outgroup). This intergroup context could enhance ingroup identification in case of a “collective” ethical climate, but could foster disengagement in case of an “individualistic” ethical climate, for instance.

Third, data collection through a self-report questionnaire could have inflated common method variance bias, although we adopted several strategies suggested by the literature in order to, at least partially, counterbalance such bias. Moreover, given that our research was cross-sectional, cause-effect relationships can be only justified by previous evidence and available theory. The size of our sample represents a further limitation that needs to be taken into account: Future research may therefore focus on confirming the pattern of relations we presented here with a larger and more heterogeneous sample.

Finally, an other intriguing question that could be addressed in future studies is whether different components of identification are involved in the relation between ethical climate and employees’ reactions. In fact, researchers highlighted the multidimensional nature of organizational identification (see, for instance, Ellemers et al., 2004), acknowledging that it expresses self-definition via cognitive and affective ties with the employing organization (Kreiner and Ashforth, 2004). Thus, future studies may disentangle whether both components of the organizational identification are at stake in the relation between ethical climate and employees’ reactions.

Overall, the present research is consistent with the idea that the social identity approach could be fruitfully applied to the organizational contexts. In particular, we provided evidence that even the effect of ethical climate on individual’s behavior depends upon the level of (personal vs. social) self-categorization, an early assumption that still proved to be valid and efficient in explaining group processes.

## Ethics Statement

The paper reports a study that has been conducted in accordance with APA ethical standards. The protocol was approved by the Ethical Committee of the Department of Psychology, Università della Campania “Luigi Vanvitelli” (Prot. Number 10/2017). In line with ethical standards of the 1964 Declaration of Helsinki, before taking part in the study, participants were informed about any relevant aspect of the study (e.g., methods, institutional affiliations of the researcher); they were informed of the right to refuse to participate in the study or to withdraw consent to participate at any time during the study without reprisal. They then confirmed that they understood the instructions well, verbally accepted to participate, and started filling out the anonymous questionnaire.

## Author Contributions

ALP collected and analyzed the data. SP, ALP, MB, VG, and MB developed the present research and equally contributed to this paper.

## Conflict of Interest Statement

The authors declare that the research was conducted in the absence of any commercial or financial relationships that could be construed as a potential conflict of interest.
